# A_2A_ Receptor Antagonism and Dyskinesia in Parkinson's Disease

**DOI:** 10.1155/2012/489853

**Published:** 2012-06-17

**Authors:** Micaela Morelli, Fabio Blandini, Nicola Simola, Robert A. Hauser

**Affiliations:** ^1^Department of Biomedical Sciences, University of Cagliari, Via Ospedale 72, 09124 Cagliari, Italy; ^2^CNR Institute of Neuroscience, 09042 Cagliari, Italy; ^3^Interdepartmental Research Center for Parkinson's Disease, National Neurological Institute C. Mondino, 27100 Pavia, Italy; ^4^Department of Neurology, University of South Florida, Tampa, FL 33613, USA

## Abstract

Dyskinesia, a major complication of treatment of Parkinson's disease (PD), involves two phases: induction, which is responsible for dyskinesia onset, and expression, which underlies its clinical manifestation. The unique cellular and regional distribution of adenosine A_2A_ receptors in basal ganglia areas that are richly innervated by dopamine, and their antagonistic role towards dopamine receptor stimulation, have positioned A_2A_ receptor antagonists as an attractive nondopaminergic target to improve the motor deficits that characterize PD. In this paper, we describe the biochemical characteristics of A_2A_ receptors and the effects of adenosine A_2A_ antagonists in rodent and primate models of PD on L-DOPA-induced dyskinesia, together with relevant biomarker studies. We also review clinical trials of A_2A_ antagonists as adjuncts to L-DOPA in PD patients with motor fluctuations. These studies have generally demonstrated that the addition of an A_2A_ antagonist to a stable L-DOPA regimen reduces OFF time and mildly increases dyskinesia. However, limited clinical data suggest that the addition of an A_2A_ antagonist along with a reduction of L-DOPA might maintain anti-Parkinsonian benefit and reduce dyskinesia. Whether A_2A_ antagonists might reduce the development of dyskinesia has not yet been tested clinically.

## 1. Adenosine A_2A_ Receptor Localization and Biochemistry

Adenosine A_2A_ receptors are present in medium to high concentrations in several basal ganglia (BG) nuclei and may therefore be capable of influencing motor activity by acting at different BG levels. This feature renders A_2A_ receptors particularly attractive for modulation of dopamine receptor functions in a disease such as Parkinson's disease (PD), which is caused by degeneration of dopaminergic neurons in the nigrostriatal pathway, but associated with changes at several receptor levels. An interesting peculiarity of A_2A_ receptors is their selective localization in the indirect striatonigral GABAergic pathway, which contains enkephalin (ENK) and which is known to lead to inhibition of motor behavior [1, 2].

A_2A_ receptors are positively coupled to adenylate cyclase and, either at the level of second messengers or through the formation of receptor heterodimers, negatively influence dopamine D_2_ receptor activity [3–6]. On the basis of this anatomical and functional organization, A_2A_ receptors acting in concert with D_2_ and D_1_ receptors are capable of affecting planning and execution of movements [7, 8]. Moreover, the low levels of A_2A_ receptors expressed in brain areas other than the BG are at the basis of the low incidence of nonmotor side effects observed in clinical trials so far performed [9]. A_2A_ receptors, however, are expressed in some peripheral organs and blood cells, underlying the importance of evaluating these elements in clinical trials testing the efficacy of A_2A_ receptor antagonists in PD [10, 11].

Interestingly, an abnormal increase in A_2A_ signaling, in the striatum of 6-hydroxydopamine- (6-OHDA-) lesioned rats, and in 1-methyl-4-phenyl-1,2,3,6-tetrahydropyridine- (MPTP-) treated primates, as well as in PD patients chronically treated with L-DOPA [12–14], might produce a prevailing tone of A_2A_ receptors, the activation of which inhibits motor activity. Therefore, blockade of the A_2A_ receptor inhibitory tone could be one of the factors underlying the positive effects produced by A_2A_ antagonists in PD.

## 2. Adenosine A_2A_ Receptor Antagonists in Animal Models of Dyskinesia

### 2.1. Behavioral Studies

Preclinical behavioral investigations suggest that A_2A_ antagonists may be of interest in the management of dyskinesia in PD. The first preclinical evidence suggesting that A_2A_ antagonists may be utilized in patients rendered dyskinetic by L-DOPA was obtained in 6-OHDA unilaterally lesioned rats subchronically treated with L-DOPA [17]. In this paradigm, the repeated administration of L-DOPA causes a progressive, sensitized, increase in contraversive turning behavior, which is thought to reproduce some aspects of the abnormal motor responses induced by the prolonged treatment with L-DOPA [17–19]. Of great interest, sensitization in contraversive turning behavior did not take place when L-DOPA was administered at a low dose in association with an A_2A_ antagonist [17, 20]. Subsequent studies utilizing a full effective L-DOPA dose in rats with established dyskinesia [21] did not report any benefit, since L-DOPA treatment alone or in combination with an A_2A_ antagonist presented the same degree of dyskinesia. These results demonstrated that A_2A_ antagonists are not antidyskinetic drugs; however, in this model, they did not worsen existing dyskinesia while increasing the efficacy of L-DOPA on motor symptoms.

Studies in MPTP-treated primates, the best experimental model of PD and PD-associated dyskinesia, have confirmed the beneficial effects of blockade of A_2A_ receptors. A_2A_ antagonists were found not to be prodyskinetic drugs, since their administration to Parkinsonian primates with established dyskinesia induced by L-DOPA relieved motor impairment and did not worsen dyskinesia [22–24]. Moreover, an attenuation of dyskinesia induced by long-term apomorphine was observed when the drug was administered in combination with an A_2A_ antagonist [25], suggesting that A_2A_ antagonists might lower the dyskinetic potential of dopamine-replacement therapy in specific conditions. The previous coadministration of an A_2A_ antagonist was also found to delay the onset of severe dyskinesia when the same primates were maintained on apomorphine alone [25].

### 2.2. Biochemical Studies

**Figure 1:**
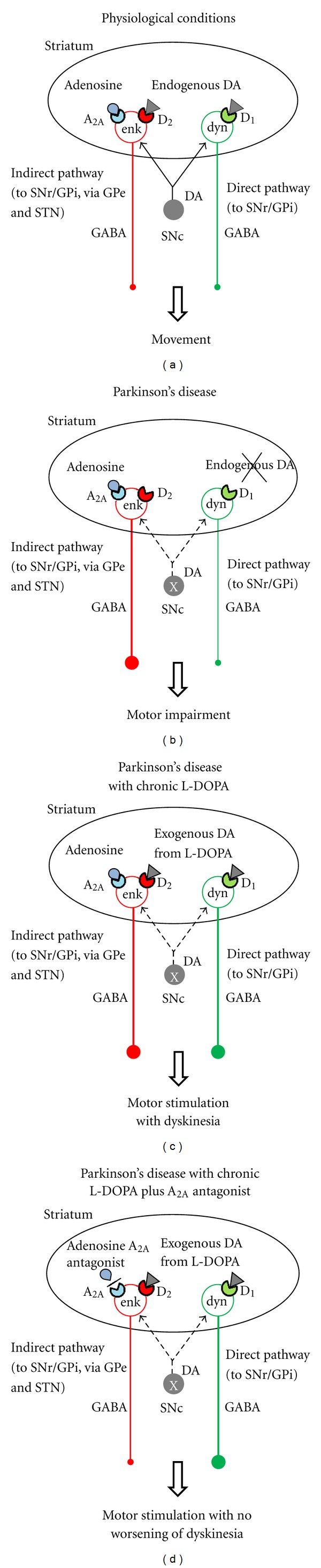
Role of A_2A_ receptors on modifications in the activity of the striatal efferent pathways. Under physiological conditions (a), striatal neurons receive dopaminergic inputs from the substantia nigra *pars compacta* (SNc). Endogenous dopamine (DA) activates the neurons belonging to the so-called direct pathway (in green), which send GABAergic projections to the substantia nigra *pars reticulata*/globus pallidus *pars interna* (SNr/GPi) and express D_1_ stimulatory dopamine receptors, together with the neuropeptide dynorphin (dyn). At the same time, dopamine also depresses the neurons belonging to the so-called indirect pathway (in red) which send GABAergic projections to the SNr/GPi via globus pallidus *pars externa* (GPe) and subthalamic nucleus (STN) and express D_2_ inhibitory dopamine receptors and the neuropeptide enkephalin (enk). Adenosine A_2A_ receptors stimulate the indirect pathway where they are selectively expressed, and their activation negatively modulates the function of D_2_ receptors. A balanced level of activity of the direct and indirect pathways is at the basis of the correct processing of motor information and movement execution. In Parkinson's disease (b), the degeneration of the neurons located in the SNc leads to a drop in the dopaminergic input to the striatum. This results in a reduced activation of the direct pathway and in a disinhibition of the indirect pathway, which is associated with the elevation of A_2A_ receptor transmission. Such unbalanced activity of the striatal output pathways is at the basis of the motor impairment observed in Parkinson's disease (b). Administration of L-DOPA restores the compromised dopaminergic tone since it stimulates the direct pathway and inhibits the indirect one (not shown). However, chronic treatment with L-DOPA (c) leads to the overactivation of the direct pathway, which together with the increase of A_2A_ receptor activity [12, 15, 16] and enhanced indirect pathway transmission is at the basis of L-DOPA-induced dyskinesia and loss of efficacy. The addition of an A_2A_ antagonist to L-DOPA (d) although not counteracting the overactivity of the direct pathway (dyskinesia) stabilizes the activity of the indirect pathway, resulting in motor stimulation, potentially without a worsening of dyskinesia.

Regarding the mechanisms underlying dyskinesia and the effects of A_2A_ antagonists in experimental models of dyskinesia, it seems likely that these drugs interfere with the neuroplastic changes induced by dopamine-replacement therapy in the dopamine-denervated BG (Figure 1). Studies in 6-OHDA-lesioned rats demonstrate that striatal dopamine denervation is associated with persistent modifications in the levels of the neuropeptides dynorphin (DYN) and ENK, as well as the enzyme glutamic acid decarboxylase 67 (GAD-67) [21, 26–28] (Figure 1). Moreover, it was observed that chronic treatment with L-DOPA, which induces a dyskinetic-like motor response, further contributes to these biochemical changes [21, 26, 27] (Figure 1). Importantly, the coadministration of an A_2A_ antagonist, besides resulting in a stable motor response, attenuated the effects of chronic L-DOPA treatment on ENK and GAD-67 [21, 26, 27]. It has to be acknowledged that, as of today, no evidence supports a direct role of DYN, ENK, and GAD-67 in dyskinesia. Nevertheless, changes in the expression of neuropeptides are a marker of the activity of striatal neurons [26]. Therefore, it can be suggested that A_2A_ antagonists modulate the effects of L-DOPA and mitigate the neuroplastic changes this drug induces in the striatum. These effects could arise from the opposite functional interactions involving adenosine A_2A_ and dopamine D_1_ and D_2_ receptors [29]. These interactions, by amplifying dopaminergic signaling, would regulate the activity of striatal output neurons in conditions of dopamine denervation and nonphysiological stimulation of dopamine receptors (Figure 1).

It has to be considered that A_2A_ receptor antagonists, in addition to their potential effects on biochemical and functional changes induced by dopamine-replacement therapy, potentiate the motor-activating effects of L-DOPA and dopaminergic agonists, allowing their use at lower, nondyskinetic doses [7]. Hence, the sparing of dopaminomimetic drugs in combination with an A_2A_ antagonist may contribute to the attenuation, or delay, of the maladaptive modifications in striatal function which underlie dyskinesia.

### 2.3. Role of Glutamate Transmission

Besides the facilitation of dopamine transmission, other mechanisms have been proposed to underlie, or at least participate in, the effects of A_2A_ receptor antagonists observed in experimental models of dyskinesia. Neuroanatomical studies demonstrate that striatal A_2A_ receptors are highly expressed at the postsynaptic level in asymmetric synapses, where they can interact with glutamate receptors [30]. Glutamate receptors are thought to participate in the pathophysiology of dyskinesia [31] and, interestingly, chronic administration of L-DOPA to 6-OHDA-lesioned rats was reported to induce a hyperphosphorylation state of the *α*-amino-3-hydroxy-5-methyl-4-isoxazole-propionic acid (AMPA) receptor [25, 32]. This effect was found to be significantly attenuated when L-DOPA was administered in combination with an A_2A_ antagonist [25, 32]. Evidence also exists that A_2A_ receptors may regulate the conductance of N-methyl-D-aspartate (NMDA) receptors [33]. This may have important implications for dyskinesia, since NMDA receptors play a major role in neuroplasticity phenomena [34, 35], including those which take place in motor circuits, and may underlie abnormal motor responses to dopamine-replacement therapy used in PD.

Interactions between A_2A_ receptors and type 5 metabotropic glutamate receptors (mGlu5) have also been reported [36–38]. In the light of the evidence showing that antagonism of mGlu5 receptors may reduce dyskinesia in MPTP-lesioned primates treated with L-DOPA [39], it is possible to envision that combined antagonism on the two receptors might contribute to the beneficial effects of A_2A_ antagonists on dyskinesia. Additional mechanisms involved in the modulation of therapy-induced abnormal motor responses by A_2A_ antagonists could include interaction with nondopaminergic and nonglutamatergic receptors, such as cannabinoid and serotonin receptors, and regulation of neurotransmitter release [40–42]. In this regard, it has to be recalled that A_2A_ receptors powerfully modulate extracellular concentrations of glutamate [43, 44], the excessive increase of which plays a role in the abnormal functioning of BG existing in PD and in neuroplasticity phenomena.

## 3. Biomarkers and Neuroimaging Studies Involving the A_2A_ Receptor

A crucial need in the translation from preclinical studies to clinical trials is the availability of reliable biomarkers, which would give the opportunity of monitoring the effects of the compound on its biological target—the adenosine receptor—in addition to evaluating its clinical efficacy. In this field, substantial contributions have been made by neuroimaging studies, while biological findings in peripheral tissues have opened interesting perspectives.

### 3.1. Neuroimaging Studies in Humans

Neuroimaging techniques, based on positron emission tomography (PET), have been recently used to analyze A_2A_ receptor distribution in the human brain, either in normal subjects or in PD patients exposed to L-DOPA; in this latter case, the purpose was to draw potential *in vivo* correlations between changes in A_2A_ receptor availability and the presence of L-DOPA-induced dyskinesias.

In 2007, Mishina et al. examined the distribution of A_2A_ receptors in the brain of 5 normal subjects using PET tracer [7-methyl-^11^C]-(E)-8-(3,4,5-trimethoxystyryl)-1,3,7-trimethylxanthine ([^11^C]TMSX) [45]. Various brain regions were examined, including the cerebellum, brainstem, thalamus, head of caudate nucleus, anterior and posterior putamen, frontal lobe, temporal lobe, parietal lobe, occipital lobe, and posterior cingulate gyrus. Results showed the highest levels of A_2A_ receptor binding in the putamen, followed by the caudate nucleus and thalamus, while the lowest levels were detected in the cerebral cortex. Using a different A_2A_ receptor-specific radiotracer, [^11^C]SCH442416, Brooks et al. assessed binding of vipadenant (3-(4-amino-3-methylbenzyl)-7-(2-furyl)-3H-[1,2,3]triazolo[4,5-d]pyrimidine-5-amine), a selective nonxanthine A_2A_ receptor antagonist synthesized by Vernalis Plc (also known as BIIB014 or V2006) [15]. Displacement of the PET tracer by increasing doses of vipadenant (2.5–100 mg/day for 10 or 11 days) was investigated in various brain regions—including the putamen, caudate nucleus, nucleus accumbens, thalamus of both hemispheres, and cerebellum—of 15 healthy volunteers. The estimated receptor occupancy of vipadenant in the brain varied from 74% to 94% at the lowest daily dose (2.5 mg), with the highest value being observed in the putamen and the lowest value in the cerebellum. Saturation was reached in all regions at the highest dose administered (100 mg). It is noteworthy that double-blind, placebo-controlled, phase 2 clinical trials with vipadenant have been conducted in PD patients, showing modest anti-PD activity, until a review of preclinical toxicology studies, conducted by Vernalis Plc, led to discontinuation of this drug in July 2010 (http://www.vernalis.com/media-centre/latest-releases/2010-releases/584/).

Recently, Mishina et al., using PET with [^11^C]TMSX, measured the binding ability of striatal A_2A_ receptors in 9 untreated PD patients, 7 PD patients with dyskinesia, and 6 age-matched control subjects [16]. They found that the distribution volume ratio of A_2A_ receptors in the putamen was larger in patients with L-DOPA-induced dyskinesias than in control subjects and that L-DOPA treatment tended to increase the presence of A_2A_ receptors in the putamen.

Further information on the relationship between A_2A_ receptors and L-DOPA-induced dyskinesias has been provided by Ramlackhansingh et al., who investigated adenosine A_2A_ receptor availability in the caudate and putamen of PD patients with (*n* = 6) and without L-DOPA-induced dyskinesias (*n* = 6) and in age-matched healthy controls (*n* = 6) [46]. In line with previous studies [12], they found that A_2A_ receptor binding was higher in the caudate and putamen of PD patients with L-DOPA-induced dyskinesias, with respect to both PD patients without L-DOPA-induced dyskinesias and controls, thereby lending further support to the view that A_2A_ antagonists may prove beneficial in the management of motor complications associated with L-DOPA treatment. It is worth mentioning that although their cohort was small and the power was probably too limited to detect a difference, Ramlackhansingh et al. did not find a correlation between striatal [11C]SCH442416 uptake and dyskinesia severity.

An additional study tested the hypothesis that blockade of striatal A_2A_ receptors, caused by the selective antagonist SYN115, a benzothiazole derivative, may reduce the inhibitory output of the striatofugal indirect pathway [47]. For this purpose, the authors used a perfusion magnetic resonance imaging (MRI) technique, which gives a functional measure of the cerebral blood flow (CBF) reflecting neuronal activity. The study was conducted during a randomized, double-blind, placebo-controlled, crossover study with SYN115 in 21 PD patients on L-DOPA. The results showed that SYN115 produced a dose-dependent decrease in thalamic CBF, which the authors deemed consistent with reduced pallid-thalamic inhibition via the indirect pathway [47].

### 3.2. Peripheral Expression of Adenosine Receptors

To our knowledge, only one paper has reported the characterization of adenosine receptors in peripheral tissues (peripheral blood cells) of human Parkinsonian subjects [48]. In this study, Varani et al. investigated affinity and density of A_1_, A_2A_, A_2B_, and A_3A_ receptors in lymphocyte and neutrophil membranes from PD patients and healthy control subjects; they also analyzed A_2A_ receptors density in autoptic samples of putamen from PD patients and control subjects. They found that A_2A_ receptors were significantly different between PD patients and controls, in terms of affinity and density, while no changes seemed to affect A_1_, A_2B_, or A_3A_ receptors. In particular, increased density of A_2A_ receptors, coupled with decreased affinity, was detected in lymphocyte and neutrophil membranes of PD patients, with respect to control subjects. This finding was associated with a reduction in the mRNA of A_2A_ receptors, while no changes were observed in the mRNAs of the other adenosine receptor subtypes investigated. The postmortem study confirmed this result, showing increased A_2A_ receptor density in the putamen of PD patients [48].

## 4. Clinical Trials of A_2A_ Receptor Antagonists 

### 4.1. Istradefylline

Clinical trials of A_2A_ antagonists in patients with motor complications have focused on reductions in OFF time rather than changes in dyskinesia. Istradefylline was the first A_2A_ receptor antagonist to enter clinical trials seeking an indication in PD. Bara-Jimenez et al. conducted an early proof-of-principle study using intravenous L-DOPA infusions in 15 moderate to advanced PD patients with motor fluctuations, 6 of whom had L-DOPA-induced peak-dose dyskinesia [49]. Twelve subjects were randomized to istradefylline, 3 to placebo, and 1 dropped out. Istradefylline 40 or 80 mg had no effect on Parkinsonian signs or dyskinesia when added to an optimal L-DOPA infusion. However, when added to a low-dose L-DOPA infusion, istradefylline 40 mg improved Unified Parkinson's Disease Rating Scale (UPDRS) motor scores by 24% (*P* < 0.05) and istradefylline 80 mg improved motor scores by 36% (*P* < 0.05). The anti-Parkinsonian response to a low-dose L-DOPA infusion plus istradefylline 80 mg was similar to an optimal-dose L-DOPA infusion. Notably, the severity of dyskinesia with a low-dose L-DOPA infusion plus istradefylline 80 mg was 45% less (*P* < 0.05) than with an optimal-dose L-DOPA infusion. This suggests that by lowering the L-DOPA dose and adding istradefylline, one might be able to maintain anti-Parkinsonian benefit and reduce dyskinesia, a paradigm that has not been studied in clinical trials using oral L-DOPA preparations.

Istradefylline was then studied in a 12-week, randomized, placebo-controlled exploratory trial in which patients with *both* motor fluctuations and dyskinesia were randomized to the addition of placebo (*n* = 29), istradefylline in ascending doses up to 20 mg/day (*n* = 26), or istradefylline in ascending doses up to 40 mg/day (*n* = 28) [50]. Anti-Parkinsonian medications were kept unchanged except that the total daily L-DOPA dose could be reduced, if necessary, to ameliorate L-DOPA-related adverse events. Over the course of the study, there were no significant changes in daily L-DOPA doses comparing istradefylline and placebo groups (*P* = 0.96). Diary results showed that the combined istradefylline groups experienced a reduction in OFF time of 1.2 hours, whereas the placebo group experienced an increase in OFF time of 0.5 hours (*P* < 0.004). Multiple assessments of change in dyskinesia did not demonstrate significant differences between the placebo and istradefylline groups, including Goetz dyskinesia scale scores (−0.2 versus −0.1, *P* = 0.3), Parkinson dyskinesia scale scores (−1.4 versus −1.3, *P* = 0.9), and UPDRS items 32–34 (−0.03 versus −0.4, *P* = 0.8). However, diary results indicated that ON time with dyskinesia was significantly more increased with istradefylline than placebo (0.6 hours versus −1.5 hours, *P* = 0.001). Troublesome dyskinesia was not included as a diary category in this study. As an adverse event, increased dyskinesia was reported by 13.8% of placebo patients and 16.7% of istradefylline patients.

This is an important study in that it is the largest study of an A_2A_ receptor antagonist in a population of patients, all of whom have L-DOPA-induced dyskinesia. In addition, dyskinesia was most thoroughly assessed by multiple scales. Clearly, the addition of istradefylline to a stable antiparkinson regimen did not reduce dyskinesia, nor was there a very substantial increase. Perhaps the most parsimonious interpretation is that overall severity of dyskinesia was essentially unchanged, but much or all of the reduction in OFF time was replaced by an increase in ON time with dyskinesia.

Subsequent trials in moderate to advanced PD patients all included subjects with motor fluctuations, some of whom had dyskinesia and some of whom did not. These included two phase 2 istradefylline trials. In one, istradefylline 40 mg/day reduced OFF time compared with placebo by 1.2 hours (*P* = 0.005) [51]. ON time with dyskinesia increased by 1.0 hour more in the istradefylline group than the placebo group (*P* = 0.035). Of this differential increase in ON time with dyskinesia, approximately 0.8 hours were ON time with nontroublesome dyskinesia (*P* = 0.065), and 0.2 hours were ON time with troublesome dyskinesia (*P* = 0.347). Dyskinesia was reported as an adverse event in 15.2% of placebo subjects and 30.2% of istradefylline subjects. In the other phase 2 study [52], istradefylline 20 mg/day reduced OFF time by 0.64 hours, and istradefylline 60 mg/day reduced OFF time by 0.77 hours (overall *P* value = 0.065). Compared with placebo, istradefylline 20 mg/day increased ON time with dyskinesia by 0.54 hours, and ON time with troublesome dyskinesia by 0.06 hours; istradefylline 60 mg/day increased ON time with dyskinesia by 0.23 hours and ON time with troublesome dyskinesia by 0.04 hours. Dyskinesia was reported as an adverse event in 14.3% of placebo subjects, 23.9% of istradefylline 20 mg/day subjects, and 22.6% of istradefylline 60 mg/day subjects.

In a phase 3 study, istradefylline 20 mg/day reduced OFF time compared with placebo 0.7 hours (*P* = 0.03) [53]. Increases in dyskinesia were similar in placebo and istradefylline groups (ON time with dyskinesia: 0.5 versus 0.7 hours, *P* = 0.57; ON time with nontroublesome dyskinesia: 0.4 versus 0.4 hours, *P* = 0.82; ON time with troublesome dyskinesia: 0.2 versus 0.3 hours, *P* = 0.48). However, dyskinesia was reported as an adverse event in 22.6% of istradefylline subjects compared with 12.2% of placebo subjects.

In a phase 3 study in Japan [54], istradefylline 20 mg/day reduced OFF time compared with placebo by 0.65 hours (*P* = 0.013) and 40 mg reduced OFF time compared with placebo by 0.92 hours (*P* < 0.001). Compared with placebo, istradefylline 40 mg/day significantly increased ON time with troublesome dyskinesia (0.35 hours, *P* = 0.011). As an adverse event, dyskinesia was reported in 2.5% of placebo subjects, 8.5% of istradefylline 20 mg/day subjects, and 6.4% of istradefylline 40 mg/day subjects.

Thus, in most of the clinical trials, the addition of istradefylline was associated with some increase in ON time with dyskinesia, and dyskinesia was reported as an adverse event more commonly in istradefylline- than placebo-treated subjects.

A recent population pharmacokinetic-pharmacodynamic study analyzed data from 1798 patients participating in 6 phase 2/3 istradefylline trials [55]. The analysis predicted a maximum probability of experiencing dyskinesia as an adverse event sometime during a study as 15.4% for placebo, 22.5% for istradefylline 20 mg/day, 24.1% for istradefylline 40 mg/day, and 24.3% for istradefylline 60 mg/day.

Thus, clinical data to date do not provide evidence for an antidyskinetic effect of istradefylline but rather suggest that istradefylline mildly increases dyskinesia in a dose-dependent fashion. Results vary slightly from trial to trial and may depend, in part, on the percentage of subjects with dyskinesia at baseline and the severity of their dyskinesia. Other potential factors may include concomitant medications such as amantadine and dietary intake of caffeine, a nonspecific adenosine antagonist, although these factors have not been systematically evaluated.

### 4.2. Preladenant

Preladenant was evaluated in a phase 2, 12-week, dose-finding study of PD patients experiencing motor fluctuations [56]. In this study, patients were randomized to preladenant 1, 2, 5, or 10 mg twice daily (BID) or matching placebo. OFF time was significantly reduced compared with placebo in subjects randomized to preladenant 5 mg BID (1.0 hours, *P* = 0.0486) and preladenant 10 mg BID (1.2 hours, *P* = 0.019). In the 5 mg BID group, compared with placebo, ON time with dyskinesia was increased 0.9 hours (*P* = 0.185), ON time with nontroublesome dyskinesia was increased by 1.0 hour (*P* = 0.064), and ON time with troublesome dyskinesia was decreased by 0.1 hours (*P* = 0.812). In the preladenant 10 mg BID group, compared with placebo, ON time with dyskinesia was increased by 1.3 hours (*P* = 0.054), ON time with nontroublesome dyskinesia was increased by 1.1 hours (*P* = 0.047), and ON time with troublesome dyskinesia was increased by 0.2 hours (*P* = 0.540). These results appear to be similar to some of the istradefylline findings in which much of the reduction in OFF time was replaced by ON time with nontroublesome dyskinesia. Dyskinesia was reported as an adverse event by 13% of placebo subjects, by 9% of preladenant 5 mg BID subjects, and by 13% of preladenant 10 mg BID subjects. This result may be different from what has been observed with istradefylline where dyskinesia was rather consistently reported more frequently as an adverse event in istradefylline-compared with placebo-treated groups. Thus, preliminary results suggest that like istradefylline, preladenant does not reduce dyskinesia, but it remains to be seen whether preladenant causes less dyskinesia than istradefylline, as suggested by these adverse event results.

To our knowledge, there have been no clinical trials evaluating whether an A_2A_ receptor antagonist can reduce the development of dyskinesia when administered in early disease concomitant with the introduction of dopaminergic therapy. Based on animal model data, this remains an important avenue for future investigation. Similarly, we are not aware of clinical trials of patients with L-DOPA-induced dyskinesia to determine whether lowering the L-DOPA dose and adding an A_2A_ receptor antagonist will allow maintenance of the anti-Parkinsonian response with reduction of dyskinesia.

## 5. Conclusions

The management of PD is most complex in the treatment of late, complicated PD, when the response to L-DOPA is associated with dyskinesia. From the studies described in the present paper, it is suggested that the management of the first (uncomplicated) phase has important consequences on the induction of dyskinesia that characterize the second (complicated) phase.

Preclinical studies suggest that A_2A_ antagonists might reduce the development of dyskinesia, but this has not yet been tested clinically. In PD patients, once dyskinesias are established, adding an A_2A_ antagonist to a stable dopaminergic therapeutic regimen does not appear to provide an antidyskinetic response, and most clinical trials have suggested a mild increase in dyskinesia in association with a reduction in OFF time. Limited clinical data suggest the possibility that in PD patients with established dyskinesia, one might be able to maintain the anti-Parkinsonian response and reduce dyskinesia by adding an A_2A_ antagonist and lowering the L-DOPA dose, but this remains to be proven. Thus, critical aspects of the potential benefits of A_2A_ antagonists with regard to dyskinesia are yet to be evaluated. 

## Abbreviations

AMPA: *α*-amino-3-hydroxy-5-methyl-4-isoxazole-propionic acid
BG: Basal ganglia
BID: Bis in die, twice a day
CBF: Cerebral blood flow
PD: Parkinson's disease
6-OHDA: 6-Hydroxydopamine
MPTP: 1-Methyl-4-phenyl-1,2,3,6-tetrahydropyridine
DYN: Dynorphin
ENK: Enkephalin
GAD-67: Glutamic acid decarboxylase 67
mGLU5: Type 5 metabotropic glutamate receptors 5
MRI: Magnetic resonance imaging
NMDA: N-Methyl-D-aspartate
PET: Positron emission tomography
UPDRS: Unified Parkinson's disease rating scale.
